# 

**Published:** 1838-07

**Authors:** 


### CONTENTS OF No. XI.

OF THE

### British and Foreign Medical Review.

JULY, 1838.

---

### Part First.—Analytical and Critical Reviews.

PAGE

Art. I.—Transactions of the Provincial Medical and Surgical Association. (Statistical and Topographical Papers.) . . . . 1

Art. II.—Mémoires de la Société Médicale d'Observation de Paris . . 28
Memoirs of the Medical Society of Observation, of Paris
1. Recherches sur l'Emphyseme des Poumons. Par M. Louis. (Researches on Emphysema of the Lungs. By M. Louis.) . . . ib.
2. Recherches sur le Cœur et le Système artériel chez l'Homme. Par J. Bizot, de Genève, m.d. &c. (Researches on the Heart and the Arterial System in Man. By J. Bizot, m.d.) . . . . . . 41
3. Essai sur quelques Points de l'Histoire de la Cataracts. Par Th. Maunoir. (Essay on certain Points in the History of Cataract. By M. Maunoir.) . 50
4. Mémoire analytique sur l'Orchite blenorrhagique. Par M. Marc d'Espine. (Analytical Essay on blennorrhagic Orchitis. By M. M. d'Espine.) . 59

Art. III.—Guy's Hospital Reports. No. V. October, 1837. Edited by G. H. Barlow, m.a. and l.m., and J. P. Babington, m.a. &c. . . 60
1. Observations on the Ganglionic Enlargement of the Pneumo-gastric Nerve; the probable Function of that Ganglion; and the Position which it occupies in the Human Subject, and in several of the lower Animals. By Mr. Cock . ib.
2. On the Distribution and probable Function of the Laryngeal Nerves. By Mr. John Hilton . . . . . . 61
3. Observations and Experiments on the Lungs of New-born Children, in relation to Medical Jurisprudence. By Mr. Alfred Taylor . . 62
4. Cases by Mr. Bransby Cooper . . . . . . 66
5. On the Diagnosis of Organic Diseases of the Uterus. By Dr. Ashwell . 71
6. Observations on Abdominal Tumours and Intumescence. By Dr. Bright . 72
7. On the Influence of Electricity as a Remedy in certain Convulsive and Spasmodic Diseases. By Dr. Addison . . . . . . ib.
8. Cases of Disease in the Fœtus. By Mr. T. W. King . . . . 74
9. Description of the Sacculus or Pouch in the Human Larynx. By Mr. Hilton ib.
10. Ophthalmic Cases treated by Mr. Morgan . . . . . ib.
11. Experiments and Observations on Albuminous Fluids. By Dr. Babington . ib.

Art. IV.—Ueber Virilescenz und Rejuvenescenz thierischer Körper. Ein Beitrag zur Lehre von der regelwidrigen Metamorphose organischen Körper. Von Dr. Carl Wilhelm Mehliss, praktischen Artze, &c. in Liebenwerda . 75
On the Virilescence and Rejuvenescence of Animals: a Contribution to the Knowledge of the regular Metamorphoses of Organic Bodies. By Dr. Charles William Mehliss, of Lebenswerda

Art. V.—1. The Cyclopædia of Practical Surgery.—Art. Abortion, by Dr. Ryan 81
2. The Cyclopædia of Practical Medicine and Surgery.—Article Abortion, by Dr. Dewees . . . . . . . ib.
3. A Dictionary of Practical Medicine, &c.—Art. Abortion, by Dr. Copland . ib.
4. The Cyclopædia of Practical Medicine.—Art. Abortion, by Dr. Lee . ib.
5. Dictionnaire de Médecine et de Chirurgie pratiques. (Dictionary of Practical Medicine and Surgery.)—Art. Avortement, by Prof. Dugès . . ib.
6. Encyclopädisches Wörterbuch der medicinischen Wissenschaften. (Encyclopædic Dictionary of Medical Science.)—Art. Abortus, by Dr. von Siebold ib.

PAGE

ART. VI.—Outlines of the principal Diseases of Females; chiefly for the Use of Students. By FLEETWOOD CHURCHILL, M.D. . . . 86

ART. VII.—Medicine and Surgery one Inductive Science; being an Attempt to improve its Study and Practice on a Plan in close Alliance with Inductive Philosophy, and offering, as first-fruits, the Law of Inflammation; addressed particularly to the Medical Student and the Profession, but easy and intelligible to the Public also: the whole being the Introduction and first Part of a System of Surgery. By GEORGE MACILWAIN, Fellow of the Royal Med. and Chir. Society, &c. . . . . . . 98

ART. VIII.—De la Morve et du Farcin chez l'Homme. Par P. RAYER, M.D., Médecin de l'Hôpital de la Charité, &c. . . . . 113

On Glanders and Farcy in Man. By P. RAYER, M.D. &c.

ART. IX.—Report of the Proceedings under a Brieve of Idiotcy, (Peter Duncan against David Yoolow,) tried at Coupar-Angus, 28-30 January, 1837; with an Appendix of relative Documents, and Introduction. By LUDOVIC COLQUHOUN, Esq., Advocate . . . . . 122

ART. X.—1. Traité de Diagnostic et de Séméiologie. Par P. A. PIORRY, M.D. . 128

Treatise on Diagnosis and Semeiology. By P. A. PIORRY, M.D.

2. Grundriss der Speciellen Semiotik. Nach den Quellen bearbeitet, von Dr. H. E. SUCKOW, Kreisphysicus in Jauer . . . . 147

Outlines of Special Semeiology. By Dr. H. E. SUCKOW.

ART. XI.—Inaugural Dissertation on the Influence of Climate on the Health and Mortality of the different Regions of the Globe. By A. S. THOMSON . 148

ART. XII.—Institutes of Surgery: arranged in the Order of the Lectures delivered in the University of Edinburgh. By Sir CHARLES BELL, K.G.H. F.R.S. L. & E. 154

ART. XIII.—Experiments and Observations on the Gastric Juice and the Physiology of Digestion. By WM. BEAUMONT, M.D., Surgeon in the United States' Army. Reprinted, with Notes, by ANDREW COMBE, M.D. &c. . 172

ART. XIV.—Du Lait, et en particulier de celui des Nourrices, considéré sous le rapport de ses bonnes et de ses mauvaises Qualités nutritives, et de ses Altérations. Par le Dr. AL. DONNÉ . . . . 181
Considerations on Milk, and particularly that of Nurses, more especially in regard to its good or bad Nutritive Qualities, and the Changes to which it is subject. By Dr. AL. DONNÉ.

ART. XV.—Practical Observations on the Preservation of Health and the Prevention of Diseases: comprising the Author's Experience on the Disorders of Childhood and Old Age, on Scrofula, and on the Efficacy of Cathartic Medicines. By Sir ANTHONY CARLISLE, F.R.S. &c. . . . 186

---

### PART SECOND.—Bibliographical Notices.

ART. I.—The Hunterian Oration, delivered in the Theatre of the Royal College of Surgeons in London, February 14, 1838. By BENJAMIN TRAVERS, F.R.S. 191

ART. II.—VELPEAU's Anatomy of Regions. Translated from the French. By HENRY HANCOCK, Esq. . . . . . 193

ART. III.—Medical Portrait Gallery. Biographical Memoirs of the most celebrated Physicians, Surgeons, &c. who have contributed to the advancement of Medical Science. By THOMAS JOSEPH PETTIGREW, F.R.S . . 194

ART. IV.—First Lines of Physiology. By DANIEL OLIVER, M.D. . . 195

ART. V.—On the Improvement of Medicine. By THEOPHILUS THOMPSON, M.D. . 196

ART. VI.—Ueber die in der Belgischen Armee herrschende Augenkrankheit: als Commentar zu Professor Dr. J. C. Juengken's Schrift über denselben Gegenstand. Von Dr. BURKARD EBLE, k. k. Regimenstfeldarzte, &c. . 197

On the Ophthalmia in the Belgian Army: a Commentary on Professor J. C. Juengken's Memoir on the same subject. By Dr. BURKARD EBLE.

ART. VII.—A new and familiar Treatise on the Structure of the Ear, and on Deafness. By A. W. WEBSTER . . . . . 199

ART. VIII.—De Nervo Faciali. Auctore Doctore MASSALIEN . . 200

Dissertation on the Facial Gland. By Dr. MASSALIEN.

PAGE

ART. IX.—A Dissertation on the Causes and Effects of Disease, considered in reference to the Moral Constitution of Man. By HENRY C. BARLOW, M.D. . 201

ART. X.—Die vergleichende Osteologie des Schläfenbeins. Zur vereinfachung der herschenden Ansichten bearbeitet. Von ED. HALLMANN . . ib.

The Comparative Anatomy of the Temporal Bone, &c. By ED. HALLMANN.

ART. XI.—1. Memoranda on difficult Subjects in Anatomy and Surgery; being a Pocket Companion for Students preparing for the College of Surgeons. By ROBERT DRUITT, M.R.C.S.L. . . . . . 202

2. The Student's Companion to Apothecaries' Hall, &c. By E. OLIVER, M.R.C.S. ib.

3. Ophthalmic Memoranda, &c. By JOHN FOOTE, F.R.C.S. . . . ib.

4. Libamenta Praxeos Medicæ. By D. SPILLAN, M.D. . . . ib.

5. A Collection of Medical Formulæ. By D. SPILLAN, M.D. . . ib.

ART. XII.—A Series of Anatomical Sketches and Diagrams, with Descriptions and References. By THOMAS WORMALD, Assistant-Surgeon, &c. . . ib.

ART. XIII.—A Treatise on Hernia: comprising the Surgical Anatomy, Operative Surgery, and Treatment. By M. W. HILLES . . . 203

ART. XIV.—Handbuch der Menschlichen Anatomie. Von C. F. T. KRAUSE, M.D. 204

Manual of Human Anatomy, &c. By Dr. KRAUSE.

ART. XV.—Neurologische Beobachtungen. Von Dr. F. H. BIDDER . . ib.

Neurological Researches. By Dr. F. H. BIDDER.

---

### PART THIRD.—Selections from Foreign Journals.

### ANATOMY AND PHYSIOLOGY.

Microscopical Observations on the Anatomy of the Aqueous Capsule and System of the Crystalline Lens. By Dr. WERNECK, of Salzburg . . 205
On Reflex Motions. By Dr. A. W. VOLKMANN . . . . 211
Anatomical Observations and Remarks. By Dr. KRAUSE . . . 218
On the Relative Motions of the Globules of the Blood and Lymph in the Blood-Vessels of Frogs. By T. M. ASCHERSON . . . . 219
Absence of the Right Lung (Cyanosis) . . . . 220

### PATHOLOGY, PRACTICAL MEDICINE, AND THERAPEUTICS.

On the Atmospheric and Epidemic Constitution of Berlin in September, 1837 . 220
Case of slow Poisoning by Oxide of Zinc. By Dr. BUSSE . . . 221
On the Juice of the Root of the Elder-tree in Dropsies . . . ib.
Researches on spontaneous Perforations of the Stomach. By M. LEFEVRE . ib.
On the Use of the Subcarbonate of Iron (Ferri Sesquioxydum,) in Hooping-cough. By Dr. STEYMANN . . . . . . . 222
On the Nature and Treatment of Syphilis in Lithuania . . . 223
On large Doses of Tartar Emetic in Articular Synovial Effusions. By Dr. GIMELLE 224
On the Use of Strychnine. By M. BALLY . . . . 225
Neuralgia of the Head, produced by a Tumour in the Brain. By Prof. B. HOLST 226
On an Epidemic Phthisis Pulmonalis. By M. MENARD . . . 227
On the Occurrence of several Spontaneous Fractures of the Ribs in a Scorbutic Individual. By Dr. GOEDECHEN . . . . . . ib.

### SURGERY.

Fatally terminating Case of Phlebitis, after Venesection. By Dr. SPÖRER . 228
Luxation of the Scapular Extremity of the Clavicle, downwards. By Dr. TOURNEL 229
On the Excision of Ulcers, &c. By M. BONNET . . . . 230
New Method of reducing Dislocations of the Os Humeri. By M. MALGAIGNE . 231
On a new Method of distinguishing Cataract. By M. SANSON . . ib.
On the Development of Adhesions of Serous Membranes, &c. By M. BELMAS . 232
Reduction of an old Luxation of the Elbow. By M. MALGAIGNE . . 233
Ligature of the Carotid Artery of a Child aged Fifteen Months. By Dr. ZEIS . ib.
Abscess beneath the Pectoralis Major, communicating with the Chest. By M. BOINET 234
Successful Application of Creosote in an old Ulcer of the Vagina . . 235

### MIDWIFERY.

Report of two Cases of Pregnancy requiring Incision of the Os Uteri to allow Delivery. By Drs. BURDACH and GRUHN . . . . 235
Singular Case of Abortion . . . . . . 236
Inversion of the Uterus resembling Polypus. By M. FLEURY, jun. . . ib.
Remarkable Case of Embryotomy on account of Exostosis of the Pelvis. By Dr. KYLL 237

### MEDICAL STATISTICS.

Statistics of the Lunatic Asylum at Aversa, (Naples.) By Filippo Volpicella . 238
Statistics of Insanity in Westphalia and Saxony . . . . 240
Statistical Notice of the Cholera-Epidemic in Berlin, in 1837. By Dr. Casper . ib.
Statistics of Suicide in London. By M. Moreau de Jonnès . . 241

### FORENSIC MEDICINE.

Murder by Carbonic Acid. By M. Devergie . . . . 241
On the Absorption of Sulphuric Acid, when introduced into the Stomach. By A. Bouchardat and the late M. Cæsar Couriard . . . 244

### MATERIA MEDICA.

New Preparation of Ipecacuanha . . . . . 247

### ANIMAL CHEMISTRY.

Microscopical Researches on the Composition of the Vaccine Fluid. By M. Dubois 247
Comparative Analysis of Healthy Bile and that containing Calculi. By P. Muratori ib.
Analysis of Liquor Amnii at two different Periods of Pregnancy. By Dr. C. Vogt 248
New Analysis of the Blood. By M. Beudant . . . . . ib.
On the Presence of Urea in Dropsical Effusions. By R. Marchand . . 249

### Selections from American and Colonial Journals.

### PATHOLOGY, PRACTICAL MEDICINE, AND THERAPEUTICS.

Extraordinary Case of Electrical Excitement. By Dr. Willard Hosford . 249
On Purulent Discharges from the Bladder and Rectum. By Dr. J. Mouat . 251
Case of Extra-uterine Pregnancy. By Allan Webb, Esq. . . . 252
On the Cure of Stammering by Moral Means. By Dr. Warren . . 253

### SURGERY.

A new Treatment in a Case of Anchylosis. By Dr. Barton . . 254
Treatment of Varicose Veins in the Pennsylvania Hospital . . . 257
A Statistical Account of Fractures, treated in the Pennsylvania Hospital . 258
Elm-bark Surgery. By Dr. W. A. M'Dowall . . . . 259
Addendum . . . . . . . 260

### Part Fourth.—Selections from British Journals.

### PHYSIOLOGY.

Case of Paraplegia. By Wm Elliott, m.d. . . . . 261

### PATHOLOGY, PRACTICAL MEDICINE, AND THERAPEUTICS.

Cases of Acute Inflammation confined to the Epiglottis. By H. Marsh, m.d. . 261
On Tapping the Head in Hydrocephalus. By T. J. Conquest, m.d. . 265
Case of Empyema. By T. Ogier Ward, m.d. . . . . 266
Case of Dropsy of the Pineal Gland. By S. S. Stanley, Esq. . . 268
Account of two Cases of Bronchial Polypus: with Remarks. By John North, f.l.s. ib.
Two Cases of Hydrophobia: effect of Morphium. By John Burne, m.d. . ib.

### SURGERY.

On Creosote, as a local Application. By George Fife, m.d. . . 270
Case of Scalded Glottis: Laryngotomy. By F. E. Hicks, Esq. . . ib.
On some Effects resulting from Wounds of Nerves. By John Hamilton, l.r.c.s. 271
Case of Talipes Varus Verus, affecting both Limbs . . . 272
Remarks on Lenticular Glaucoma. By Wm. Mackenzie, m.d. . . 274
A Mode of relieving Enlargement of the Veins of the Testicle. By Mr. Wormald ib.
Treatment of Intussusception by Inflation. By S. Mitchell, Esq. . . ib.

### Part Fifth.—Medical Intelligence.

On the Present State of Medicine and Medical Education in Holland. By C. J. Nieuwenhuijs, m.d. . . . . . . 275
Sketch of the Present State of Medicine in Portugal . . . 284
The Provincial Medical and Surgical Association . . . . 287
OBITUARY—Thomas Blizard, Esq., Professor Fergus, Dr. Edw. Harrison, Rasori, Baron Desgenettes, and Dr. Deckmann . . 288–290
Books received for Review . . . . . . 291

